# Hyperplastic polyp or sessile serrated lesion? The contribution of serial sections to reclassification

**DOI:** 10.1186/s13000-020-01057-0

**Published:** 2020-12-09

**Authors:** Diana R. Jaravaza, Jonathan M. Rigby

**Affiliations:** 1 Division of Anatomical Pathology, National Health Laboratory Service, Stellenbosch University, Tygerberg Hospital, Cape Town, South Africa; 2 Department of Anatomical Pathology, Health Services Executive, Cork University Hospital, Cork, Ireland

**Keywords:** Sessile serrated lesion, Sessile serrated adenoma/polyp, Hyperplastic polyp, Levels, Serial sections

## Abstract

**Background:**

The histological discrimination of hyperplastic polyps from sessile serrated lesions can be difficult. Sessile serrated lesions and hyperplastic polyps are types of serrated polyps which confer different malignancy risks, and surveillance intervals, and are sometimes difficult to discriminate. Our aim was to reclassify previously diagnosed hyperplastic polyps as sessile serrated lesions or confirmed hyperplastic polyps, using additional serial sections.

**Methods:**

Clinicopathological data for all colorectal hyperplastic polyps diagnosed in 2016 and 2017 was collected. The slides were reviewed and classified as hyperplastic polyps, sessile serrated lesion, or other, using current World Health Organization criteria. Eight additional serial sections were performed for the confirmed hyperplastic polyp group and reviewed.

**Results:**

Of an initial 147 hyperplastic polyps from 93 patients, 9 (6.1%) were classified as sessile serrated lesions, 103 as hyperplastic polyps, and 35 as other. Of the 103 confirmed hyperplastic polyps, 7 (6.8%) were proximal, and 8 (7.8%) had a largest fragment size of ≥5 mm and < 10 mm. After 8 additional serial sections, 11 (10.7%) were reclassified as sessile serrated lesions. They were all less than 5 mm and represented 14.3% of proximal polyps and 10.4% of distal polyps. An average of 3.6 serial sections were required for a change in diagnosis.

**Conclusion:**

Histopathological distinction between hyperplastic polyps and sessile serrated lesions remains a challenge. This study has uncovered a potential role for the use of additional serial sections in the morphological reappraisal of small hyperplastic polyps, especially when proximally located.

## Introduction

Although serrated polyps have in common a saw-tooth morphology of crypts, it is important to distinguish between hyperplastic polyps (HPs) and sessile serrated lesions (SSLs) because of the inherent difference in risk of malignant transformation [1]. This risk in turn informs colonoscopic surveillance intervals [1–7].

Historically, colorectal polyps were classified as conventional adenomas (adenomatous polyps) and hyperplastic (metaplastic) polyps, with only the former being recognised as having a risk of malignant transformation [2, 6, 8, 9]. In a landmark study published in 2003, Torlakovic et al. proposed that not all hyperplastic polyps were equal, and that there was a subgroup of serrated/ hyperplastic polyps with morphological and genetic features distinct from “benign” hyperplastic polyps, that were likely neoplastic [10, 11]. They proposed the use of the term “sessile serrated adenoma” for these polyps [10]. The terms sessile serrated polyp, and sessile serrated adenoma/ polyp have been used interchangeably [6, 12].

Serrated colonic neoplasia is now classified by the World Health Organization (WHO) as; hyperplastic polyps (HPs), sessile serrated lesions (SSLs) with or without dysplasia, traditional serrated adenomas (TSAs), and serrated adenoma, unclassified [2, 13]. Sessile serrated lesions are more common in the right colon, and hyperplastic polyps in the left [13, 14]. International studies show that HPs account for 83–96%, SSLs 3–11%, and TSAs 1–7% of all serrated polyps [3]. However, there is a paucity of data on the prevalence of sessile serrated lesions in the South African population.

Approximately 60% of colorectal carcinomas (CRCs) are preceded by conventional adenomas via the classic adenoma-carcinoma sequence, 5% are attributable to Lynch syndrome, and serrated neoplasia accounts for 20–35% [3, 6, 9, 15–17]. There is strong evidence linking SSLs and TSAs to CRC, whereas hyperplastic polyps are generally considered to follow a benign course [2, 6, 8, 13, 18]. Serrated polyps can progress to CRC via the serrated neoplasia pathway [2, 8, 16, 19–21]. They have also been linked to interval carcinomas, especially proximally [6, 16, 19, 21, 22]. Misinterpretation of SSLs as HPs may contribute to this phenomenon.

There are a number of studies in which previously diagnosed hyperplastic polyps were reviewed and reclassified, in an attempt to improve diagnostic rates and understanding of serrated neoplasia [4, 8, 23–25]. Many of these have shown significant numbers being reclassified [14]. Interobserver variation in reclassification rates has been attributed in part to the non-uniform application of diagnostic criteria, and lack of awareness [21, 25]. Our search did not reveal similar published studies based on African data. In addition, some authors have advocated the routine use of additional/ deeper tissue levels to aid in improving diagnosis of colorectal polyps, whilst others have questioned their utility [13, 21, 26, 27]. As such there is no clear recommendation on how many levels or sections are adequate to improve diagnosis of SSLs.

## Materials and methods

### Study design and setting

A retrospective review of HPs from the colorectum, diagnosed at Tygerberg Academic Hospital Division of Anatomical Pathology, South Africa from 01/01/2016 to 31/12/2017 was conducted. Ethical approval was granted by the Stellenbosch University Health Research Ethics Committee.

### Participants

A search of our pathology database was performed for the period under review using SNOMED codes for ‘hyperplastic polyp’ and ‘colon’ or ‘rectum’. Demographic and clinical data were extracted from electronic reports. Suitable cases were selected using the following inclusion criteria: HP diagnosis, adult patient (age ≥ 18 years), original slides and wax blocks available, documented polyp site, and no concurrent colorectal polyposis syndrome or colorectal carcinoma.

Simultaneous histological reviews of the original haematoxylin and eosin (H&E) slides were conducted by the two investigators (JR a senior pathologist with experience in gastrointestinal pathology, and DJ a senior anatomical pathology resident). This culminated in a consensus diagnosis of confirmed HP, SSL or other.

Eight additional serial sections were performed for the confirmed HPs. Additional sections were placed in a standard sequence on slides. On further review, a final diagnosis of HP or SSL was rendered. The standard practice in our laboratory is that multiple ‘upfront’ serial/ sequential sections are cut for each colorectal polyp once the full face of the biopsy is seen. This number varies according to the polyp size, generally ranging from 4 to 8. In this study serial sections and consecutive sections will be used interchangeably. During both stages of histological review, the researchers were blinded to the polyp site.

### Diagnostic criteria

**Fig. 1:**
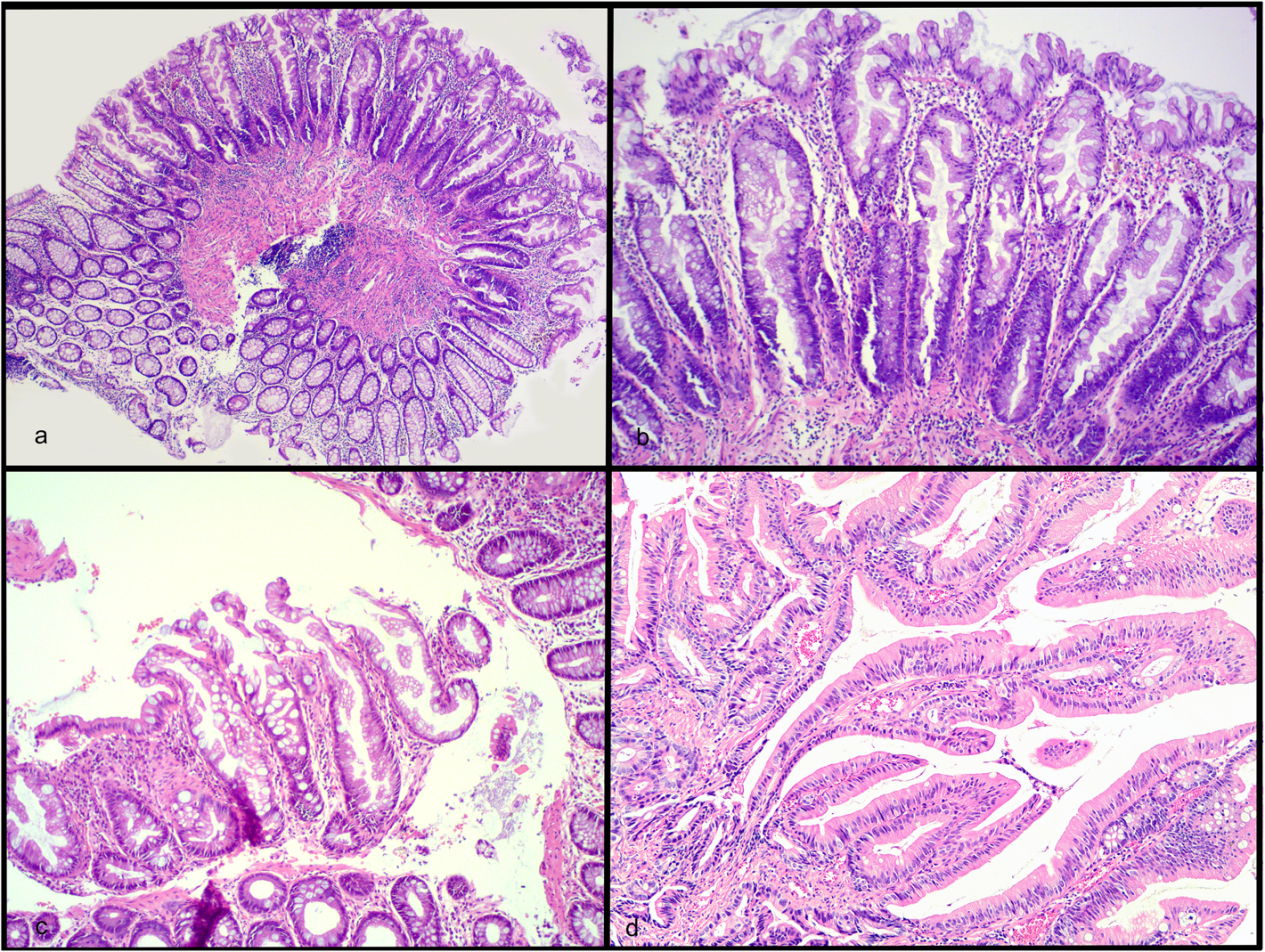
H&E stain. ×40 hyperplastic polyp (**a**), ×100 hyperplastic polyp showing superficial and crypt serration limited to the upper two-thirds (**b**)**,** × 100 sessile serrated polyp with boot- shaped crypt (**c**), × 100 traditional serrated adenoma with conspicuous eosinophilic cytoplasm, loss of mucin, and pencillate nuclei (**d**)

2019 WHO criteria for SSL were used [13]. The minimum criterion was ‘the presence of at least one *unequivocally* architecturally distorted crypt’ [13]. Features used to diagnose HPs included serrations limited to the surface epithelium and superficial crypts, proliferative zones limited to crypt bases, and evenly spaced crypts [13]. Basal dilatation, significant distortion or submucosal misplacement should have been absent [13]. See Fig. 1.

### Data analysis

Clinicopathological variables analysed were: age, gender, polyp site in relation to the splenic flexure (right- up to and including, and left- distal to), and polyp size based on the largest fragment (< 5 mm, 5–9 mm and ≥ 10 mm). These variables were compared between confirmed HPs and SSLs. The level at which the minimum diagnostic criterion was first fulfilled on additional sections was designated 1–8. Data collection and simple mathematical analysis was performed using Microsoft Excel for Windows. No statistical analysis was performed.

General pathologists as well as one pathologist with a special interest in gastrointestinal pathology were responsible for reporting during the study period.

## Results

The database search yielded 163 polyps from 150 patients, of which 147 polyps met the inclusion criteria. The remainder were unsuitable due to wrong SNOMED code, missing wax blocks, or missing original H&E slides.

**Fig. 2:**
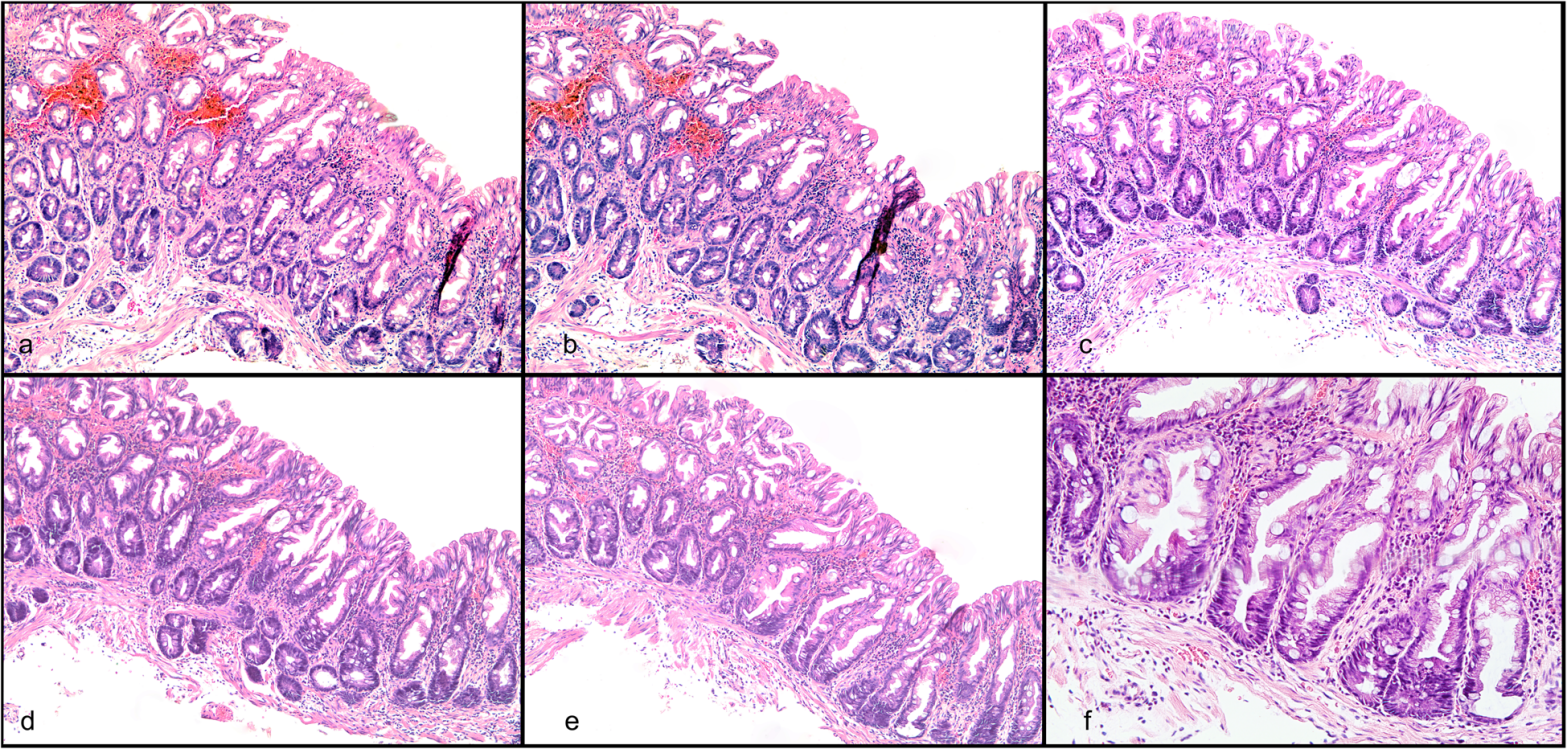
H&E stain of HP reclassified as SSL. × 100 first (**a**) and fourth (**b**) of five sections of original slides showing features of hyperplastic polyp, × 100 first of six additional sections showing hyperplastic polyp (**c**), third of additional sections showing hyperplastic polyp (**d**)**,** fifth of additional sections showing features of SSL at × 100 (**e**)**,** and ×200 (**f**)

**Fig. 3:**
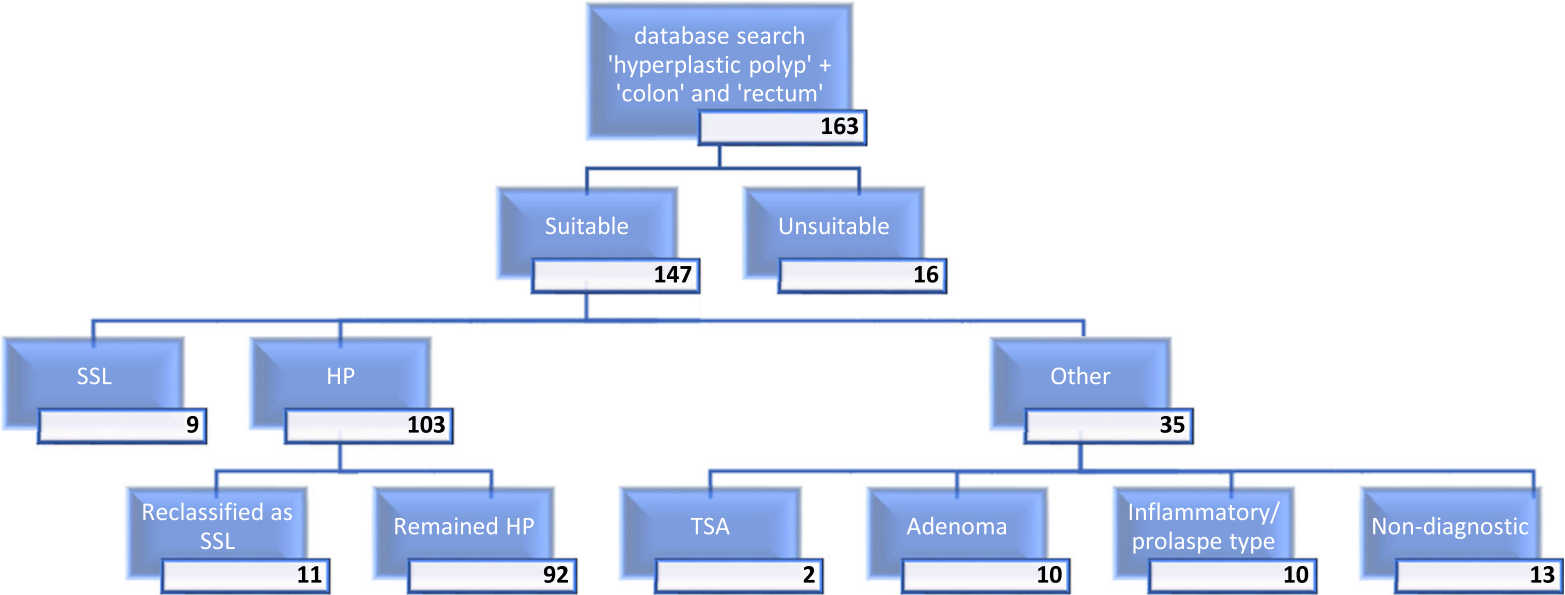
Flow diagram showing outcome of histological review of initial 163 cases

Initial review of the original slides resulted in reclassification of 9 (6.1%) of the HPs as SSLs. 55.6% of these were distal, and 77.8% were <  5 mm. The remainder were classified as confirmed HPs or other. The category ‘other’ comprised predominantly non-diagnostic normal fragments of colorectal mucosa (37%), followed by inflammatory/ prolapse-type polyps (29%), and conventional adenomas (29%). There were 2 misclassified traditional serrated adenomas. See Figs. 2 and 3.

For 93 patients, contributing to the 103 confirmed HPs, the mean and median ages were 59 and 62 years respectively, with the youngest being 23 and the oldest 81 years. Fifty-eight polyps (56.3%) were from women, 96 (93.2%) were located distally, and the majority 95 (92.2%) were <  5 mm. None were ≥ 10 mm. See Table 1. Of the 103 patients, 71 had one or more presenting complaints or indications for colonoscopy documented. The majority (75%) had one or more abdominal or lower gastrointestinal tract symptoms. Haematochezia (27%) was the most frequent of these, and anaemia the least (1.4%). Additional indications included other symptomatology such as change in bowel habits or abdominal pain, risk factors for colorectal carcinoma (32%) including inflammatory bowel disease, previous colorectal polyps or carcinoma, or a family history of colorectal carcinoma. No cases were indicated as age-related surveillance.

**Table 1:** Clinicopathological characteristics of the study population

**Age (years)**		**Reclassified to SSL**	**Remained HP**
**Mean**	59	60	59
**Median**	62	63	62
**Range**	23–81	45–76	23–81
	**Initial HPs (n, %)**	**Reclassified to SSL (n, %)**	**Remained HP (n, %)**
**Total cases**	103 (100)	11 (10.7)	92 (89.3)
**Gender**			
**Female**	58 (56.3)	5 (45.5)	53 (57.6)
**Male**	45 (43.7)	6 (54.5)	39 (42.4)
**Polyp site**			
**Right**	7 (6.8)	1 (9.1)	6 (6.5)
**Left**	96 (93.2)	10 (90.9)	86 (93.5)
**Polyp size**			
**< 5 mm**	95 (92.2)	11 (100)	84 (91.3)
**5–9 mm**	8 (7.8)	0	8 (8.7)
**≥ 10 mm**	0	0	0

Further analysis of these confirmed HPs using eight additional serial sections, resulted in reclassification of 11 (10.7%) as SSLs. All 11 were ≤ 5 mm. Although the majority of these were distally located (90.9%), as a proportion, 14.3% of proximal and 10.4% of distal HPs were reclassified. When both site and size were considered, a larger proportion of proximal polyps < 5 mm (16.7%) were reclassified as SSLs, compared to distal ones (11.2%). A mean of 3.6 additional serial sections was required to reach a change in diagnosis. See Tables 1, 2 and 3. A change in diagnosis to SSL was made if at least one of the diagnostic features was visualised on the additional sections. The most common feature was horizontal crypt growth, alone or in combination with other features.

**Table 2:** Diagnosis after performing eight additional sections

	Cases	%	Right sided		Left sided	
			Cases	%	Cases	%
**Sessile serrated lesion**	11	**10.7**	1	**14.3**	10	**10.4**
**Hyperplastic polyp**	92	**89.3**	6	**85.7**	86	**89.6**

**Table 3:** Final diagnosis in relation to polyp site and size

	Right sided (***n*** = 7)				Left sided (***n*** = 96)			
	< 5 mm		5–9 mm		< 5 mm		5–9 mm	
	Cases	%	Cases	%	Cases	%	Cases	%
**Sessile serrated lesion**	1	**16.7**	0	**0**	10	**11.2**	0	**0**
**Hyperplastic polyp**	5	**83.3**	1	**100**	79	**88.8**	7	**100**
**Total**	**6**	**100**	**1**	**100**	**89**	**100**	**7**	**100**

The mean age of patients with a final diagnosis of HP was comparable to those whose polyps were reclassified as SSLs (59 vs 60 years). However, patients with SSLs had a narrower age range 45–76 compared to the wider age range of patients with HPs (23–81 years). See Table 1.

## Discussion

Colorectal cancer is the leading cause of cancer deaths in the United States [8, 9]. According to the International Agency for Research on Cancer (IARC), 2020 report, the colorectum was the third most common cancer site in men, and the second in women [28]. The incidence rate in 2018 was higher in developed countries (e.g. in Europe), compared to developing countries (e.g. in Africa and Asia), at 20.9/100000 women in the former, and 5.9/100000 women in the latter [28]. The South Africa National Cancer Registry 2016 report indicated that colorectal carcinoma was the 6th most common histologically diagnosed malignancy in women (4.3%), and 4th most common in men (5.29%), which translate to age-standardised incidence rates of 6.81/100000 and 11.01/100000 respectively [29]. Breast carcinoma was the leading malignancy in women, and prostate carcinoma in men.

Over a two-year period, only 163 colorectal polyps were signed out as HPs. Similar studies showed sample sizes ranging from 49 to 8324 [4, 11, 20, 24, 25]. The lack of a National colonoscopic surveillance programme in South Africa may account for this seemingly low number. Abdominal and lower gastrointestinal tract symptomatology was documented in 75% of patients, whilst only 32% had recognised risk factors for colorectal carcinoma.

### Initial reclassification

6.1% of the initial 147 polyps signed out as HPs were reclassified as SSLs on the original slides using the current WHO criteria, with only one displaying dysplastic features. These cases most likely represent interobserver variability which may be attributable in the greater part to increasing knowledge and less strict criteria used currently than was applied during the review period. Using the current criteria, *at least one unequivocally* architecturally distorted crypt was sought. During the study period (2016–2017) the 2010 WHO criteria were more stringent and required at least three affected crypts [30]. A number of authors had highlighted that these older criteria were not clear-cut and the prerequisite of at least three affected crypts might not be practical [31]. Prior to the 2019 WHO edition, there was no uniformity as to which criteria were used at our centre to diagnose SSLs, with some opting for the less stringent Expert Panel recommendations, and others, the 2010 WHO [32].

23.8% of HPs were placed in the ‘other’ category which included non-diagnostic, inflammatory/ prolapse type, tubular adenoma, and TSAs. It is appreciated that prolapse mucosal type changes can mimic HPs especially in the left colon [25]. Confusion of HPs with tubular adenomas and TSAs is attributed to lack of awareness of morphology.

### Diagnostic criteria

The application of eight additional serial sections resulted in reclassification of an additional 10.7% of HPs as SSLs. None had dysplastic features. This reclassification represents a higher proportion than when we applied the current WHO criteria alone (6.1%). Reported rates of reclassification in the literature range from 2.6–85%, in studies from 2007 to 2019 [4, 11, 19, 24, 25, 33–40]. The wide range is mostly attributable to the multiple diagnostic criteria with different levels of stringency that characterised earlier years [4, 7, 23, 31]. Of the aforementioned studies, only Bettington et al., and Janjua et al. relied solely on the WHO criteria (2010), whilst Gill et al., and Khalid et al. used a combination of criteria that included reference to the WHO [23–25, 37]. The remainder relied on those of Torlakovic et al., Snover et al., and local Pathology Societies [4, 11, 33–36, 38–40]. In a systematic review and meta-analysis in 2017 by Niv et al., a reclassification rate of 17% was reported, which is comparable to this study [19]. Reported reclassification rates of more than 30%, differed from the current study in that they only assessed right sided HPs (Gill et al), only polyps larger than 5 mm (Tinmouth et al), or did not utilise consensus criteria (Khalid et al) [20, 25, 37]. Interestingly these three studies relied on a combination of diagnostic criteria which included the WHO. There were five studies reporting reclassification rates lower than ours [33, 35, 38–40]. None of these utilised the WHO criteria. Instead those of Snover et al., Torlakovic et al., the Expert Panel criteria, and the German Society of Pathology were referenced. Of these, the Expert Panel criteria were the least stringent, and closely approximate the current WHO criteria. Although current criteria are less stringent, it still appears that SSLs remain underdiagnosed [5, 13, 23]. The application of different diagnostic criteria is a well-recognised source of interobserver variability [5, 23, 31, 41]. The strict, uniform application of consensus criteria however, improves diagnostic reproducibility of SSLs [1, 42]. In addition, diagnostic criteria specify only morphology as integral to diagnosis [12].

### Additional serial sections

6.1% of SSLs were diagnosed on the original H&E levels, whilst 10.7% required an average of 3.6 additional serial sections over and above the ‘upfront’ ones. Many institutions routinely request ‘upfront’ levels on all gastrointestinal biopsies [26]. This requirement optimises the biopsy, especially when poorly orientated. Of the studies reviewed, only two enumerated the number of tissue levels examined for reclassification. Schachschal and colleagues analysed at least 8 serial sections of each polyp [39]. In a Swiss study on the other hand, the standard was three levels. In a survey of United Kingdom and North America based pathologists, Chetty et al. reported that 86% of respondents had routine levels processed for colorectal polyps, 7% reported the converse and did not see the utility in requesting these to facilitate a diagnosis, and 7% used levels (routine or additional) when considering the diagnosis of SSL [5]. Although Warnecke and colleagues specifically emphasise that deeper sections may be useful in differentiating serrated lesions, especially HPs from SSLs, they did not find any significant patient factors, endoscopic indications, or typical histological features that correlated with the yield of step sectioning [43]. We could not find literature that specifically evaluated the number of tissue levels in relation to reclassification of HPs. The general recommendation is that well orientated specimens with visible crypt bases are essential as the diagnosis of SSL relies on crypt architecture, and may consist only of focal changes [13, 21, 40]. Thus, the use of serial sections or step sections is advised, especially in poorly orientated right sided polyps, equivocal, and predominantly superficial biopsies [12, 25, 44]. In five studies investigating the utility of additional sectioning on diagnostic yield of initially non- diagnostic colonic polyps, the yield on additional sections ranged from 10 to 31.1%, with the most common diagnosis being tubular adenomas and HPs [43]. The study designs showed great disparity regarding the number of routine sections (which involved serial sections, ribbons and step sections), or melting of wax blocks and rotating of tissue. Additional tissue sections have attendant labour, financial, turnaround time, storage, loss of diagnostic tissue, and further ancillary testing implications, which must be weighed against potential diagnostic yield [26, 27, 45].

### Polyp site

Proximal polyps accounted for only 6.8% of the 103 HPs, and 9.1% of reclassified SSLs. Three previous studies also reported SSLs being more numerous distally [24, 35, 40]. However, in one of these, SSLs were still more likely to be proximally located than HPs [35]. 14.3% of proximal polyps in this study were reclassified as SSLs, in contrast to 10.4% of distal ones. This is consistent with other reports [4, 20, 34, 35, 38]. In 111 patients with polyps more than 5 mm, Tinmouth et al. found that 28.8% of polyps were reclassified, comprising 48.5% of proximal polyps and 17.3% of distal ones [20]. In a Winnipeg study, of 204 HPs, 11.8% were reclassified as SSLs, comprising 17% of proximal versus 4% of distal polyps [34]. Similarly, Farris et al. reported reclassifying 35% of proximal and 18% of distal polyps [4]. Interestingly, Kim et al. reported there being no right colon predilection for SSLs misclassified as HPs [36]. The importance of SSLs predominating in the proximal colon is that they are susceptible to being missed at colonoscopy [17]. This phenomenon is ascribed to their subtle endoscopic features, a tendency to be obscured by mucus and debris, and poorer bowel preparation proximally [8, 12, 16]. It is notable that most interval carcinomas tend to be proximal, and have been linked to preceding SSLs [3, 16, 19]. Some authors however, argue that interval carcinomas are more likely to arise from missed or incompletely resected lesions, than a specific molecular pathway [12]. Despite this proximal bias, proximal location should not be regarded as a prerequisite for diagnosis [42]. From this study, a lower threshold for serial sectioning could be suggested for proximal hyperplastic-appearing polyps.

### Polyp size

Polyp size is a recognised important clinical predictor of subsequent dysplasia and invasive carcinoma [17, 42]. A higher frequency of synchronous adenomas in patients with large and (proximal) serrated polyps, has been reported [6, 12]. In addition, large distal serrated polyps are reported to be four times as likely to be associated with a proximal adenocarcinoma [12]. Of the initial 147 HPs, 9 were reclassified as SSLs, *without* the performance of additional tissue levels. Only 2 were in the 5–9 mm range, and the rest were < 5 mm. Of the 11 polyps that were reclassified following additional serial sections, all were < 5 mm. This contrasts with most of the literature which emphasises larger size.

A meta-analysis of 2625 HPs from 8 studies, showed that only proximal location and polyp size > 5 mm was statistically significant for an association with reclassification as SSL [19]. In a study of 702 HPs, of the 188 that were reclassified, 45.7% were < 5 mm, 44.2% 5–9 mm, and 10.1% ≥10 mm [4]. However, when comparing HP polyps < 5 mm and those ≥5 mm, they found 20% of the former and 37% of the latter were reclassified as SSLs. They concluded that polyp size ≥5 mm was a predictor of reclassification as an SSL (odds ratio, 2.09; 95% confidence interval, 1.34–3.26) [4]. In a longitudinal study of SSLs, high grade lesions were more likely to develop in polyps > 5 mm, as compared to those ≤5 mm [35]. Tinmouth and colleagues reclassified only polyps > 5 mm, and they found that 41.4% were 6–9 mm, and 58.6%, ≥10 mm [20]. Some studies have disregarded polyps < 5 mm citing difficulty with orientation [11]. Others did not find size to be a significant predictor of reclassification [36, 38]. Pai et al. emphasise that diagnosis should be primarily morphological, to enable adequate risk assessment, for example with small proximal HPs [12].

The discrepancy in our study may be explained by polyp size being measured as per the largest fragment received in the laboratory. Bettington et al. are of the opinion that this method is unreliable [23]. This is controversial as others opine that using endoscopist dimensions may be subjective [4]. In addition, inconsistent documentation of endoscopic size, piecemeal resection, biopsy instead of complete resection of larger polyps, and fragmentation in transit, could also have contributed to inaccuracies in size. Conversely, in relation to our study, it may be that the difficulty with discrimination of SSL from HP at our centre, is inversely related to polyp size. The focal SSL changes in smaller polyps may require more diligent sectioning of the wax block to uncover them.

### Age and gender

There was no difference between the mean age of patients whose polyps were reclassified to SSLs (60 years), and those which remained HPs (59 years). However, the age range for reclassification was narrower, with the patients middle aged and older (45–76 years) vs (23–81 years). Similarly, Bettington et al. found the mean age for HP was 59.7 years and that for SSL was 58.6 years [23]. The literature is divided regarding age. Some authors did not find a significant relationship between reclassification and age [19, 20, 23, 33, 36]. In contrast, Schramm et al. found that age ≥ 65 years was significantly associated with reclassification [38].

54.5% of our reclassified polyps occurred in men. Schramm et al. reported an even higher frequency of SSLs in men (82.9%) [38]. Two studies highlighted that SSLs have a greater tendency to occur in women [22, 23]. However, the majority found that female gender was not a significant predictor of reclassification [19, 20, 33, 36, 40].

### Continental studies

Of the African studies reviewed, there is a low incidence of serrated polyps in general, and SSLs in particular [46]. At one of South Africa’s tertiary institutions, it was reported that 34% of patients with colorectal carcinoma had synchronous colonic polyps [47]. Of these, the majority (51%) were tubular adenomas, whilst HPs (3%), and sessile serrated adenomas (1%), were in the minority [47]. A clinical review of colonoscopy findings of 989 patients conducted between January 2008 and March 2010, in Johannesburg, South Africa revealed a majority 50.7% were tubular adenomas, without any SSLs [48]. In contrast, a review of colonoscopy records conducted in Cape Town, South Africa showed that of 246 polyps, 10.6% were hyperplastic and only 1.6% were serrated adenomas. The latter were not further subclassified [49]. In an Egyptian study, HPs accounted for 15% of non-adenomatous polyps, the majority being hamartomatous. No SSLs were reported [46]. A Nigerian study reported 30.8% inflammatory and hyperplastic polyps, but no SSLs, in a clinicopathological review of patients presenting from 2013 to 2017 [50]. Whether the low reported rates of SSLs on our continent are a true reflection of our population, related to endoscopist polyp detection rates, or a diagnostic dilemma, is uncertain.

### Strengths, limitations, and applications

The strength of this study lies in that it was conducted at a large, central referral hospital which is presumed representative of the majority population who depend on public health care. In addition, blinding to polyp location during assessment helped to avoid bias when assessing larger polyps. The single centre experience, small sample size, unorientated, sometimes fragmented specimens, submission of multiple biopsies in the same container, and lack of an expert gastrointestinal pathology consult service may have posed limitations.

There are application possibilities arising from this study such as using findings to inform the development of quality assessment tools for the optimum orientation, processing, and interpretation of colonoscopic biopsy specimens, in collaboration with the Gastroenterology department. They can also be used as a platform to discuss discrepant diagnoses of tubular adenomas and TSAs with pathologists. Other centres could conduct similar reviews to improve understanding of the prevalence of SSLs in their populations, and increasing diagnostic accuracy through familiarisation with, and uniform application of current WHO criteria.

In some Western populations the diagnosis of some SSLs informs a shorter colonoscopic surveillance interval [3, 19, 22]. The progression rate for SSLs is thought to be slower than that of adenomatous polyps [21]. An outcome of hyperplastic polyps reclassified as SSLs should translate to a shorter follow-up interval, as our institution follows the 2012 US Multi-Society Task Force on Colorectal Cancer Guidelines. As the reclassified SSLs were small, no change to clinical management was instituted for these cases. When National colonoscopic surveillance guidelines become available in our country, it is envisaged that improved diagnosis of SSLs will positively impact colorectal carcinoma prevention.

## Conclusion

The histopathological discrimination of HPs from SSLs can be difficult. This study has uncovered a potential role for the use of additional serial sections over and above ‘upfront’ levels, especially for proximal hyperplastic polyps less than 5 mm in size. In addition, familiarity with, and consistent application of current WHO diagnostic criteria for SSLs is imperative. Due to the small sample size of this study, additional research is recommended. Improved diagnostic accuracy will prevent unnecessary, expensive investigations on one hand, and interval colorectal carcinoma on the other hand.

## Data Availability

Deidentified spreadsheets of study data and ethics certificates are available on request.

## Abbreviations

CRC: Colorectal carcinoma
H&E: Haematoxylin and eosin
HP: Hyperplastic polyp
SNOMED: Systematised nomenclature of medicine
SSL: Sessile serrated lesion
TSA: Traditional serrated adenoma
WHO: World Health Organization
